# p58^IPK^ suppresses NLRP3 inflammasome activation and IL-1β production via inhibition of PKR in macrophages

**DOI:** 10.1038/srep25013

**Published:** 2016-04-26

**Authors:** Evgenii Boriushkin, Joshua J. Wang, Junhua Li, Maulasri Bhatta, Sarah X. Zhang

**Affiliations:** 1 Department of Ophthalmology/Ross Eye Institute, University at Buffalo, the State University of New York, Buffalo, NY 14214, USA; 2 SUNY Eye Institute, the State University of New York, Buffalo, NY 14214, USA; 3 Department of Biochemistry, University at Buffalo, the State University of New York, Buffalo, NY 14214, USA

## Abstract

The NLRP3 inflammasome activation is a key signaling event for activation and secretion of pro-inflammatory cytokines such as IL-1β from macrophages. p58^IPK^ is a molecular chaperone that regulates protein homeostasis through inhibiting eIF-2α kinases including double-stranded RNA–dependent protein kinase (PKR), which has been recently implicated in inflammasome activation. Herein we investigate the role of p58^IPK^ in TLR4 signaling and inflammasome activation in macrophages. Primary bone marrow-derived macrophages (BMDM) was isolated from p58^IPK^ knockout (KO) and wildtype (WT) mice and treated with lipopolysaccharide (LPS) and ATP to activate TLR4 signaling and stimulate inflammasome activation. Compared to WT macrophages, p58^IPK^ deficient cells demonstrated significantly stronger activation of PKR, NF-κB, and JNK and higher expression of pro-inflammatory genes TNF-α and IL-1β. Coincidently, p58^IPK^ deletion intensified NLRP3-inflammasome activation indicated by enhanced caspase 1 cleavage and increased IL-1β maturation and secretion. Pretreatment with specific PKR inhibitor or overexpression of p58^IPK^ largely abolished the changes in inflammasome activation and IL-1β secretion in p58^IPK^ null macrophages. Furthermore, immunoprecipitation assay confirmed the binding of p58^IPK^ with PKR, but not other TLR4 downstream signaling molecules. Collectively, these results suggest a novel and crucial role of p58^IPK^ in regulation of inflammasome activation and IL-1β secretion in macrophages.

The mammalian innate immune system recognizes and responds to different types of pathogens in order to protect the host through multiple signaling responses. The responses are initiated when pathogen-associated or damage-associated molecular patterns are recognized by specific cell surface receptors such as Toll-like receptors (TLRs) and C-type lectin receptors or cytoplasmic proteins including the retinoic acid-inducible gene (RIG)-I-like receptors and NOD-like receptors (NLRs)^1^. Upon engagement, these receptors activate a series of signaling pathways resulting in upregulation of genes encoding pro-inflammatory cytokines, such as tumor necrosis factor alpha (TNFα), interleukin 1β (IL-1β), and interleukin 6 (IL-6). These molecules orchestrate the inflammatory response and play critical roles in inducing acute-phase proteins, modifying endothelial function and permeability, recruiting immune cells to injury site, and regulating survival and cell death. While TNFα and IL-6 are regulated primarily at transcriptional and translational levels, production of IL-1β is regulated by a two-step mechanism. The first step is the expression of inactive IL-1β precursor (pro-IL-1β) induced by the TLR signaling. The second step is the cleavage of pro-IL-1β by activated caspase-1^2^. The complex that activates caspase-1, called the inflammasome, is composed of NLRs, apoptosis-associated speck-like proteins containing caspase recruitment domains (ASC) and pro-caspase-1.

Inflammasome formation is triggered by a range of endogenous and exogenous substances that emerge during infections, tissue damage, and metabolic imbalances. Once the protein complexes have formed, the inflammasomes activate caspase-1, which proteolytically activates the inactive IL-1β and IL‑18 precusors. Processed (active) IL-1β is one of the key inflammatory cytokines implicated in many human diseases including autoinflammatory syndromes^3^, diabetes^4^ and neurodegenerative disorder^5^. It has been shown to mediate pancreatic β cell destruction in type 1 diabetes and increases the risk for type 2 diabetes by inducing insulin resistance^4^. Inhibition of IL-1β signaling by IL-1 receptor antagonist IL-1RA or IL-1β antibody demonstrate encouraging therapeutic effect in type 2 diabetes^6^,^7^. Thus, understanding the regulatory mechanisms and identifying endogenous suppressors for IL-1β holds great promise for developing new treatment of inflammatory human diseases.

p58^IPK^ is a multifunctional molecular chaperone that regulates protein homeostasis. When presenting in the cytosol p58^IPK^ functions as an inhibitor of eIF-2 kinases to control protein translation^8^,^9^,^10^, while in the endoplasmic reticulum (ER) it acts as a co-chaperone and regulator of glucose-related protein 78 (GRP78) and participates in protein folding^11^. Among the eIF-2 kinases, double-stranded RNA dependent protein kinase (PKR) was the first being identified to bind to p58^IPK^ and regulate protein translation during viral infections^12^. Apart from its regulatory role in protein translation, activation of PKR was found implicated critically in regulation of the expression and production of proinflammatory cytokines through governing multiple pathways mediated by mitogen activated protein kinases (MAPK)^13^,^14^, IκB kinase (IKK)^15^, IFN-β-promoter simulator 1 (IPS-1) signaling^16^, and transcriptional factors including NF-κB and, c-Jun, and activating transcription factor 2 (ATF2)^14^,^15^,^17^. Recently, Lu and colleagues reported a novel role of PKR in coordinating the activation of inflammasomes and maturation of IL-1β^18^. Interestingly, this finding was challenged by another study showing that PKR is dispensable for inflammasome activation^19^. Nevertheless, the role of PKR and its regulation in macrophage activation and IL-1β production is intriguing.

Here we investigate the influence of p58^IPK^ as an inhibitor of PKR on TLR4 signaling, NLRP3 inflammasomes, and IL-1β production in macrophages. Using bone marrow derived macrophages (BMDM) from wild-type and p58^IPK^ knockout mice, we demonstrate that p58^IPK^ inhibits lipopolysaccharide (LPS)-induced activation of NF-κB and c-jun N-terminal kinase (JNK) and decreases the expression of pro-inflammatory cytokines TNFα, IL-1β, and IL-6. Furthermore, we show that p58^IPK^ suppresses NLRP3 inflammasome activation and reduces IL-1β production through inhibition of PKR.

## Results

### p58^IPK^ regulates LPS-induced PKR, NF-κB and JNK activation in macrophages

**Figure 1:**
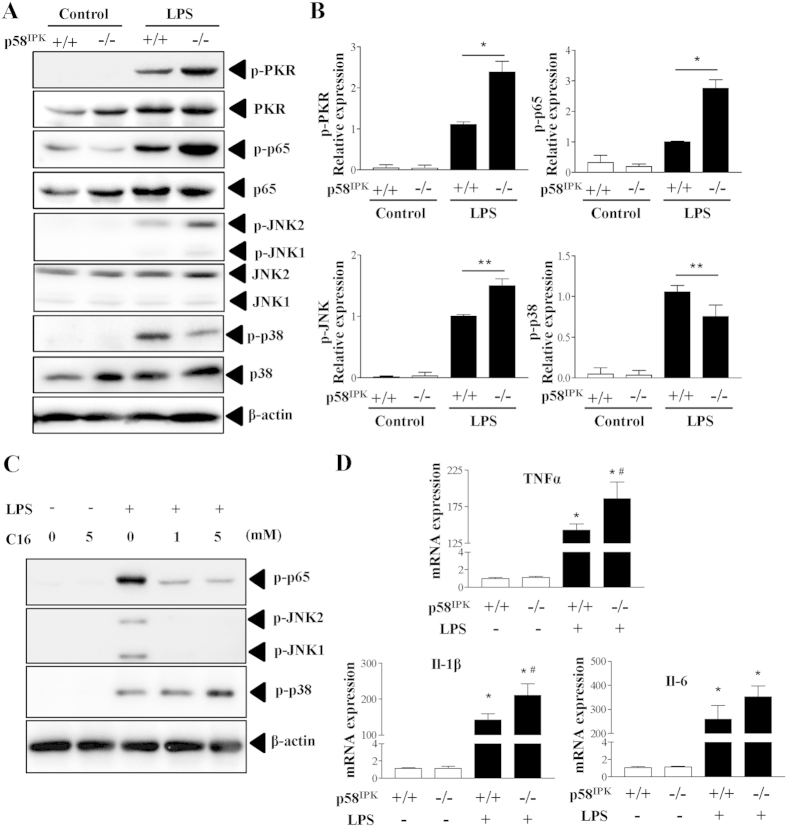
p58^IPK^ regulates LPS-induced activation of PKR, NF-κB and JNK MAPK pathways. (**A,B**) BMDM isolated from WT (p58^IPK+/+^) or p58^IPK^ knockout (p58^IPK−/−^) mice were cultured and treated with LPS for 15 min. Phosphorylation of PKR, NF-Κb p65, JNK, and p38 MAPK was evaluated by western blot analysis and quantified by densitometry. β-actin was used as a loading control. Data represent means ± SD from at least 3 independent experiments. *P < 0.05 and **P < 0.01. (**C**) BMDM from WT mice were pretreated with specific PKR inhibitor C-16 and then exposed to LPS or vehicle for 15 min. Whole cell lysates were subjected to western blot analysis. (**D**) BMDM from WT (p58^IPK+/+^) or p58^IPK^ knockout (p58^IPK−/−^) mice were treated with LPS for 2 h. mRNA expressions of TNFα, IL-1β and IL-6 were determined by real-time qPCR and normalized by 18S. Data represent means ± SD of 3 independent experiments. *P < 0.05 vs. no LPS treatment, ^#^p < 0.05 vs LPS-treated p58^IPK+/+^ cells.

Recent studies have implicated PKR in TLR4-dependent signaling in mouse cells^20^,^21^. To determine whether p58^IPK^ as an inhibitor of PKR is involved in regulation of TLR4 pathway in macrophages, we isolated BMDM from WT and p58^IPK^ knockout mice and exposed the cells to LPS to activate TLR4 signaling. We found that LPS treatment for 15 min induced markedly increased phosphorylation of PKR, NF-κB p65, and JNK in WT (p58^IPK+/+^) BMDM, and to a significantly greater extent in p58^IPK^-deficient (p58^IPK−/−^) cells (Fig. 1A,B). Interestingly, LPS induced a less increase in the activation of p38 MAPK in p58^IPK−/−^ BMDM compared to controls. To determine whether PKR is a key mediator of these changes, BMDM were pretreated with specific PKR inhibitor C13H8N4OS (C16). We found that inhibition of PKR largely abolished LPS-induced phosphorylation of NF-κB p65 and JNK but augmented p38 MAPK activation (Fig. 1C). These results indicate that the effect of p58^IPK^ on TLR4 signaling is likely mediated by regulation of PKR.

Since TLR4 downstream transcription factors NF-κB and AP-1 critically regulate several proinflammatory genes, including TNFα^22^, IL-1β^23^ and IL-6^24^, we evaluated the role of p58^IPK^ in regulation of proinflammatory gene expression in LPS-treated macrophages. Compared to WT controls, LPS induced a significantly higher increase in TNFα and IL-1β expression in BMDM of p58^IPK−/−^ (Fig. 1D). In contrast, p58^IPK^ deletion led to a modestly higher increase in IL-6 expression, which, however, did not differ significantly from the WT control.

### p58^IPK^ deficiency exacerbates NLRP3 inflammasome activation

**Figure 2:**
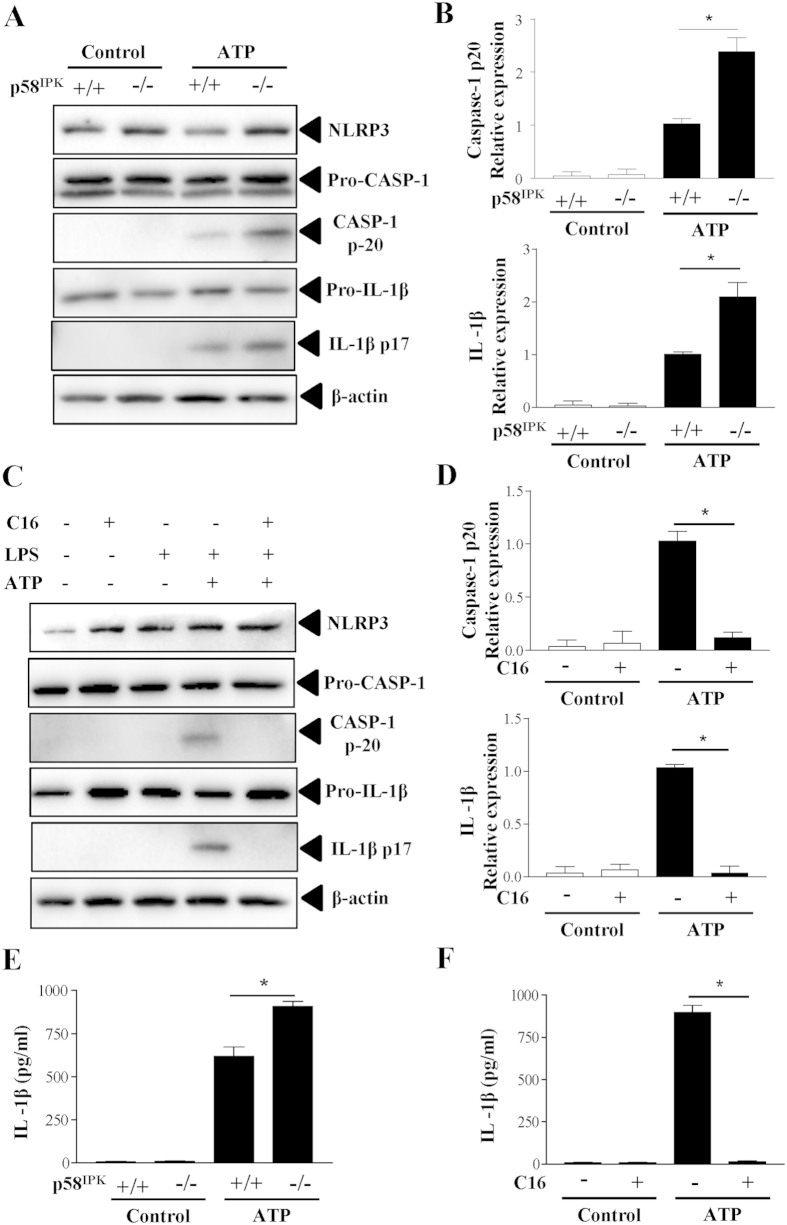
p58^IPK^ deficiency exacerbates NLRP3 inflammasome activation via PKR. (**A,B**) BMDM from WT (p58^IPK+/+^) or p58^IPK^ knockout (p58^IPK−/−^) mice were primed with LPS (500 ng/ml) for 4 h and then stimulated with ATP (5 mM) for 1 h. Protein levels of NLRP3, pro- and cleaved capase-1 and IL-1β were determined by western blot analysis and quantified by densitometry. Data represent means ± SD of 3 independent experiments. *P < 0.01. (**C,D**) BMDM from p58^IPK^ knockout (p58^IPK−/−^) mice were pretreated with C16 and then exposed to LPS and ATP to induce inflammasome activation. Whole cell lysates were assessed by western blot analysis. Protein levels were quantified by densitometry and normalized by β-actin. Data represent means ± SD of at least 3 independent experiments. *P < 0.01. (**E,F**) Levels of IL-1β secreted into the medium of p58^IPK+/+^ and p58^IPK−/−^ BMDM cultures were measured by ELISA. Data represent means ± SD of at least 3 independent experiments. *P < 0.01.

Next, we explored the role of p58^IPK^ in the regulation of NLRP3 inflammasome activation in macrophages. To induce inflammasome activation, BMDM were primed with ultra-pure LPS and then stimulated with ATP. Inflammasome activation was evaluated by cleavage of caspase-1 and maturation of IL-1β. Compared to the WT control, p58^IPK−/−^ macrophages show significantly higher levels of cleaved caspase-1 and mature IL-1β (Fig. 2A,B). The levels of pro-caspase-1 and pro-IL-1β as well as NLRP3, did not differ in p58^IPK−/−^ and control macrophages.

PKR activation has been shown to play a crucial role in inflammasome activation^18^. To elucidate whether increased NLRP3 inflammasome activation in p58^IPK−/−^ macrophages was attributed to enhanced PKR activation, we isolated BMDM from p58^IPK−/−^ mice and pretreated the cells with PKR inhibitor C16 prior to inducing inflammasome activation. We found that inhibition of PKR completely blocked LPS-induced caspase-1 activation and IL-1β maturation (Fig. 2C,D). Furthermore, we measured the level of IL-1β secreted from macrophages into the media. Coincident with the changes in inflammasome activation, IL-1β secretion was significantly increased in p58^IPK−/−^ BMDM and the increase in IL-1β secretion was completely abolished in cells pretreated with C16 (Fig. 2E,F).

### Overexpression of p58^IPK^ reduces NLRP3 inflammasome activation and IL-1β secretion

**Figure 3:**
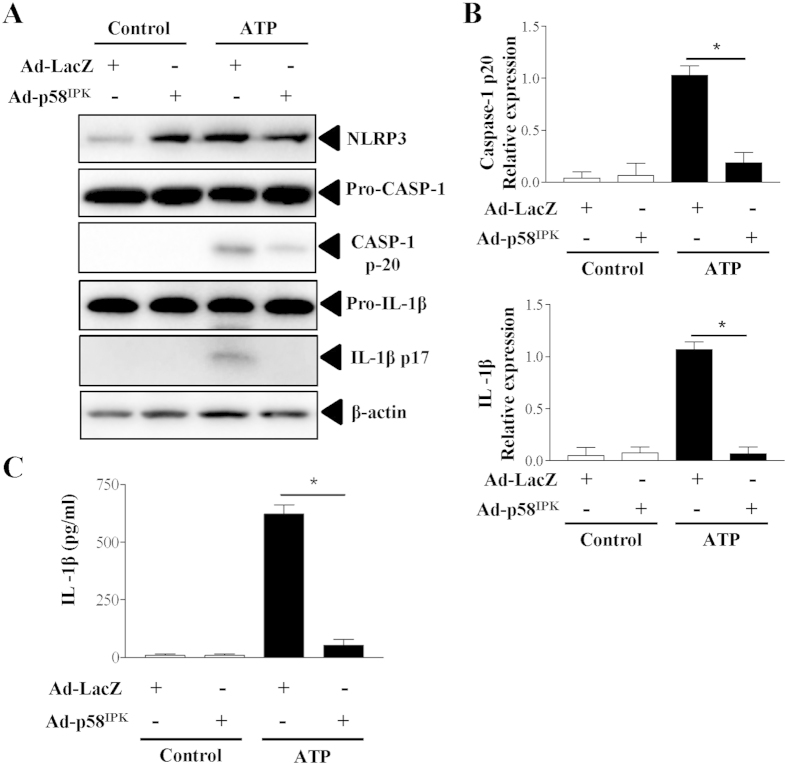
Overexpression of p58^IPK^ reduces inflammasome activation and IL-1β secretion. (**A,B**) BMDM from WT mice were transduced with Ad-p58^IPK^ or Ad-LacZ at MOI of 50 for 24 h. Cells were then treated with LPS and ATP to induce inflammasome activation. Whole cell lysates were subjected to western blot analysis. Protein levels were quantified by densitometry and normalized by β-actin. Data represent means ± SD of 3 independent experiments. *P < 0.01. (**C**) Levels of IL-1β secreted into the medium of BMDM were measured by ELISA. Data represent means ± SD of 3 independent experiments. *P < 0.01.

To further evaluate the effect of p58^IPK^ on activation of NLRP3 inflammasomes, we overexpressed p58^IPK^ by adenovirus (Ad-p58^IPK^) in BMDM isolated from WT mice. Adenovirus expressing β-galactosidase (Ad-LacZ) was used as a control. After 24 h, adenoviral transduced cells were primed with LPS and then stimulated with ATP to activate inflammasomes. As shown in Fig. 3A,B, overexpression of p58^IPK^ significantly reduced caspase-1 activation and IL-1β maturation, suggesting an inhibition in inflammasome activation. Moreover, p58^IPK^ overexpression drastically reduced IL-1β secretion from macrophages (Fig. 3C), further confirming an inhibitory role of p58^IPK^ in inflammasome activation.

### p58^IPK^ does not alter the adhesion capacity of LPS-activated macrophages to endothelial cells

**Figure 4:**
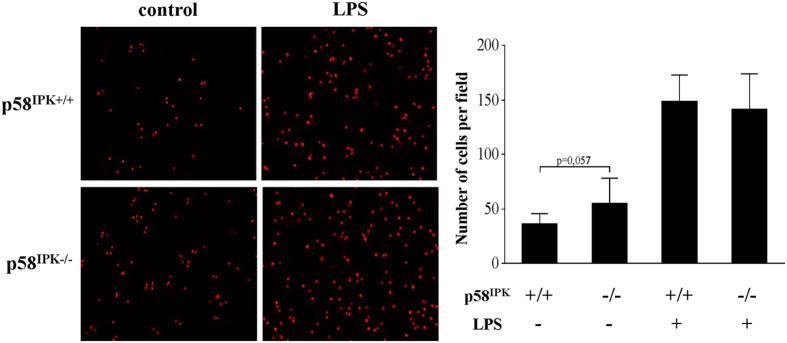
p58^IPK^ deficiency does not alter macrophage adhesion to endothelial cells. BMDM from WT (p58^IPK+/+^) or p58^IPK^ knockout (p58^IPK−/−^) mice were treated with LPS (250 ng/ml) or vehicle for 6 h. Cells were labeled with fluorescent dye PKH26 and then added to HUVECs for adhesion assay. (**A**) Representative images of showing BMDM (red) adhering to HUVECs. LPS treatment increased BMDM adhesion to endothelial cells. However, no difference was observed in LPS-treated p58^IPK−/−^ macrophages compared to p58^IPK+/+^ controls. (**B**) Quantification of adhered BMDM to HUVECs.

Macrophage adhesion and interaction with endothelial cells is considered a key event in the pathogenesis of vascular injury in diabetic complications^25^. We determined whether deficiency of p58^IPK^ results in overactivation of macrophages thereby enhancing macrophage adhesion to endothelial cells. BMDM from p58^IPK−/−^ and WT mice were treated with 250 ng/ml LPS or vehicle for 6 h, labeled with fluorescence dye, and resuspended for adhesion assay. As shown in Fig. 4, deficiency of p58^IPK^ did not result in significant change in adhesion of LPS-stimulated BMDM to HUVECs. Non-treated p58^IPK−/−^ macrophages has a tendency to increase adhesion but the difference was not significant.

### p58^IPK^ interacts with PKR in macrophages

**Figure 5:**
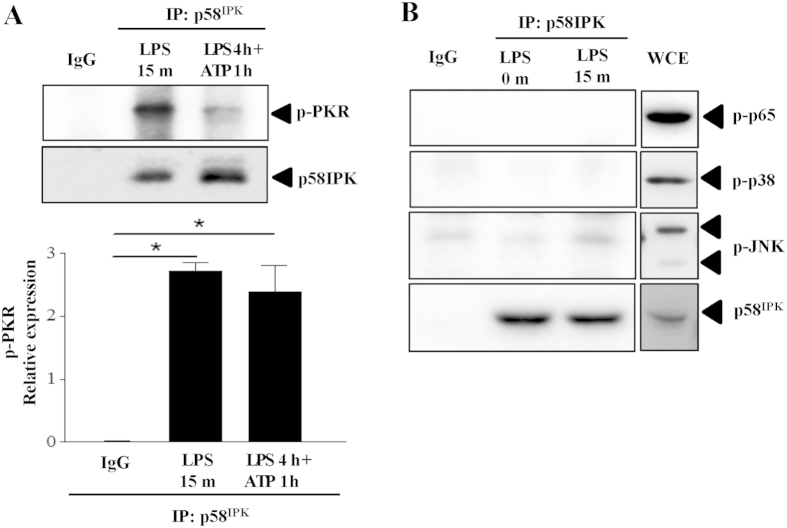
Interaction between p58^IPK^ and PKR in BMDM. (**A**) Mouse BMDM were treated as indicated and harvested. Cell lysate was subjected to immunoprecipitation using antibody against p58^IPK^ or non-immunized serum (IgG) as a negative control, followed by western blot analysis. Relative protein levels of PKR were determined by densitometry. Data represent means ± SD of 3 independent experiments. (**B**) No positive binding of p58^IPK^ with NF-κB p65, p38 or JNK was revealed in BMDM treated with LPS for 15 min, but strong activation of each molecule was observed in the whole cell extract (WCE) from cells under the same treatment. The gels were run under the same experimental conditions, and full-length blots are presented in Supplementary data.

To determine whether p58^IPK^ interacts with PKR and TLR4 downstream signaling molecules in macrophages, we performed immunoprecipitation to explore protein binding in BMDM treated with LPS for 15 min when significant activation of TLR4 signaling molecules was observed, or in cells with inflammasome activation. We found that p58^IPK^ was successfully coimmunoprecipitated with p-PKR in both treatment groups (Fig. 5A). In contrast, we did not observe association of p58^IPK^ with other signaling molecules including p-NF-κB p65, p-p38 and p-JNK (Fig. 5B). These results further confirmed that the inhibitory effect of p58^IPK^ on TLR4 signaling and inflammasome activation is mediated by its regulation of PKR.

## Discussion

**Figure 6:**
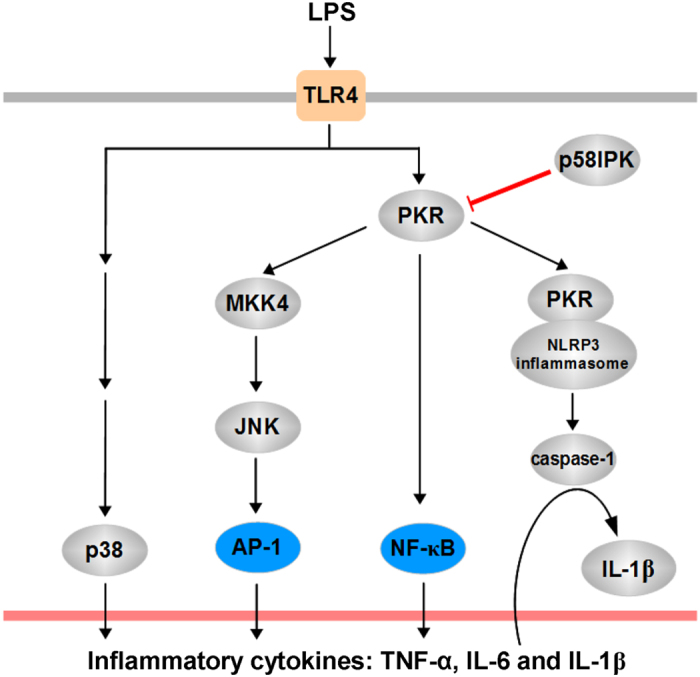
Schematic summary of the role of p58^IPK^ in regulation PKR-dependent signaling and proinflammatory cytokine production.

p58^IPK^ is a key regulator of protein translation^8^,^9^,^10^ and protein folding^11^ depending on its subcellular localization^26^. In addition, p58^IPK^ suppresses the activity of PKR and regulates the host innate defense response during viral infection^27^. Although the function of p58^IPK^ in protecting against cell death is well characterized, its role in macrophage-derived proinflammatory cytokine production and inflammatory response remain elusive. In the present study we investigated the potential role of p58^IPK^ in the regulation of pro-inflammatory gene expression and inflammasome activation in macrophages. Our results provide strong evidence that p58^IPK^ functions as a potent inhibitor of inflammasome activation and IL-1β production in macrophages. Furthermore, we demonstrate that p58^IPK^ suppresses TLR4 downstream signaling pathways of NF-κB and JNK and these effects are mediated by inhibition of PKR (Fig. 6).

PKR is a well-established regulator of innate immune response to viral infection playing an important role in host cell survival and viral amplification. Recently activation of PKR has been linked to chronic inflammation pertinent to metabolic disorders such as diabetes^28^,^29^. Interestingly, the role of PKR in inflammatory signaling pathways appears to vary in different cell types according to prior studies^20^,^21^,^30^. In mouse embryonic fibroblasts, PKR regulates the activity of p38 and JNK MAPKs in response to LPS and pro-inflammatory cytokines^20^. In contrast, a study using BMDM reported that the PKR is dispensable for activation of p38 and JNK MAPKs or activation of NF-κB^30^. Yet, recent work by Cabanski and colleagues shows that in alveolar macrophages PKR is critical for the activation of NF-κB and JNK, but not p38 MAPK, pathways^21^. Using BMDM isolated from p58^IPK^ knockout mice and pharmacological PKR inhibitor, we demonstrated that deletion of p58^IPK^ augments PKR, NF-κB p65 and JNK MAPK activation, but reduces p38 activation, while inhibition of PKR by specific pharmacological inhibitor largely abolished NF-κB p65 and JNK, but not p38 MAPK activation. These results support that manipulating PKR by either endogenous or exogenous inhibitors differentially regulates p38 and JNK MAPKs and NF-κB p65 activation in macrophages. Consistent with these results, we found that deficiency of p58^IPK^ exacerbated LPS-induced expression of TNF-α and IL-1β, but did not alter the induction of IL-6 in macrophages. Future investigation is needed to evaluate the effect of p58^IPK^ on TNF-α and IL-6 protein production and secretion from macrophages.

In addition to regulation of canonical inflammatory pathways, recent studies provide compelling evidence that supports a new and potential role of PKR in inflammasome activation^31^. Initial work by Lu and associates^18^ suggests that PKR physically interacts with inflammasome components including NLRP3, NLRP4, and absent in melanoma 2 (AIM2). Furthermore, autophosphorylation of PKR in a cell-free system with recombinant NLRP3, ASC and pro-caspase-1 is sufficient to reconstitute inflammasome activity, suggesting that PKR is a potent inducer of inflammation activation. Thus, identifying the endogenous inhibitors for PKR-mediated inflammasome activation could lead to new treatment approaches to reduce inflammation and mitigate inflammasome-related cell death. Our major findings in this study have identified p58^IPK^ as a strong inhibitor of PKR signaling and inflammasome activation. Deletion of p58^IPK^ significantly intensified LPS-induced inflammasome activation and IL-1β secretion, which was completely eliminated by pharmacological inhibition of PKR. Furthermore, we show that overexpression of p58^IPK^ significantly reduced IL-1β maturation and secretion. Interestingly, we found that the adhesion capacity of LPS-activated macrophages was not affected by p58^IPK^ deficiency, suggesting a relative specific role of p58^IPK^ on inflammasome activation and IL-1β secretion. Given the important role of IL-1β in broad inflammatory diseases, manipulating p58^IPK^ may serve as a new therapeutic target in controlling signaling pathways of IL-1β production and inflammation.

In summary, our study demonstrates an important role of p58^IPK^ in regulation of PKR-mediated TLR4 downstream NF-κB and JNK MAPK pathways, inflammasome activation, and IL-1β secretion from macrophages. Therefore, enhancing the function of p58^IPK^ could lead to a new approach to prevent inflammasome and IL-1β-related tissue injury and pathological consequences such as neurodegeneration.

## Methods

### Materials

Ultrapure LPS was purchased from Sigma Laboratories (St. Louis, MO, USA). NLRP3 inflammasome agonist ATP was obtained from InvivoGen (San Diago, CA, USA). Fetal bovine serum, Dulbecco’s modified eagle’s medium (DMEM), 1 × MEM on-essential amino acids, 1 × MEM vitamins, and sodium bicarbonate were obtained from Gibco laboratories (Grand Island, NY, USA). RIPA buffer, inhibitor mixture, PMSF, and sodium orthovanadate were purchased from Santa Cruz Biotechnology (Santa Cruz, CA, USA).

### Mice

p58^IPK^ knockout (KO) mice were kindly provided by Dr. Michael G. Katze (University of Washington). All methods were carried out in accordance with the ARVO Statement for the Use of Animals in Ophthalmic and Vision Research. All experimental protocols were approved by the Institutional Animal Care and Use Committees at the University at Buffalo, State University of New York.

### Isolation, culture and adenoviral transduction of mouse bone marrow derived macrophages (BMDM)

Mouse BMDM were isolated from bone marrow and cultured as described previously^32^,^33^. To activate TLR4 signaling and induce inflammatory gene expression, cells were treated with LPS (250 ng/ml) for up to 24 h. To induce inflammasome activation, cells were primed with LPS (500 ng/ml) for 4 h and then stimulated with ATP (5 mM) for 1 h^18^. In experiments with overexpression of p58^IPK^, BMDM were transduced with adenoviruses expressing p58^IPK^ (Ad-p58^IPK^) or β-galactosidase (Ad-LacZ) as control at a multiplicity of infection (MOI) of 50 as described previously^34^. Twenty-four hours after transduction, cells were treated LPS and ATP to induce inflammasome activation.

### Western blot analysis

Cells were lysed and sonicated in radioimmunoprecipitation (RIPA) buffer with protease inhibitor mixture, PMSF, and sodium orthovanadate. Protein concentration was measured by BCA protein assay (Thermo scientific, Rockford, IL, USA). The samples were resolved by SDS-PAGE and transferred to nitrocellulose membrane and blotted with specific antibodies: anti-p58^IPK^, anti-p-NF-κB, anti-NF-κB, anti-p-p38, anti-p38, anti-p-JNK, anti-JNK, (Cell Signaling Technology, Boston, MA, USA), anti-p-PKR, anti-PKR, anti-β-actin (Santa Cruz Biotechnology, Santa Cruz, CA, USA), anti-IL-1β (R&D Systems, Inc., Minneapolis, MN, USA), anti-caspase-1 (p-20), and anti-NLRP3 (AdipoGen, Inc, San Diego, CA, USA). The immunoblots were developed using chemiluminescence (SuperSignal West Dura Extended Duration Substrate, Thermo Scientific, USA) and visualized under Chemidoc MP Imaging System (Bio-Rad, Hercules, CA, USA).

### Quantitative Real-time RT–PCR

Total RNA was extracted using an E.Z.N.A. total RNA kit I (Omega bio-tek, Georgia, GA) following the manufacturer’s instructions. The quantity of total RNA was determined by spectrophotometry using the Synergy HT BioTek (Winooski, Vermont, USA). A Maxima First Strand cDNA synthesis kit (Thermo Scientific, Grand Island, NY) was used for cDNA synthesis. Real-time RT-PCR was performed using SYBR Green PCR Master Mix (Bio-Rad Laboratories, Hercules, CA). 18S ribosomal RNA was chosen as endogenous control to normalize quantification of target genes. The primer sequences are as follows: mouse TNFα (forward, CGG TGCCTATGTCTCAGCCT; reverse, TTGGGCAGATTGACCTCAGC); mouse IL-1β (forward, CAGGCAGGCAGTATCACTCA; reverse, GAGGATGGGCTCTTCTTCAA); mouse IL-6 (forward, AGTCCGGAGAGGAGACTTCA; reverse, TTGCCATTGCACAACTCTTT).

### IL-1β ELISA

The protein levels of IL-1β in cell culture supernatants were measured using IL-1β ELISA kits (R&D Systems) according to the manufacturer’s instructions.

### Immunoprecipitation

Antibodies against the p58^IPK^ were used to precipitate proteins from cell lysate in the presence of 20 μl protein A/G beads (Santa Cruz) overnight at 4 °C. Protein complexes were washed five times with RIPA buffer, and then incubated at 95 °C for 3–5 minutes and resolved by western blotting.

### Macrophage adhesion assay

Macrophage adhesion assay was carried out using human umbilical vein endothelial cells (HUVEC) and primary mouse BMDM. Briefly, HUVECs were cultured to confluence. BMDM were isolated and cultured for 8 days. Before adhesion assay, BMDM were washed three times with serum-free RPMI medium 1640 and labeled with dye PKH26 (Sigma Aldrich). Approximately 1 ml of 20,000 BMDM were incubated with HUVEC for 20 min. Unadhered cells were then washed out three times with serum-free RPMI medium 1640. The adherent cells were counted in 6–8 randomly selected optical fields in each well as described previously^25^. Fluorescence microphotographs of the cells were taken under an Olympus microscope (Tokyo, Japan).

### Statistical analysis

The quantitative data were expressed as mean ± SD. Statistical analyses were performed using an unpaired student’s t-test when comparing two groups and one-way analysis of variance (ANOVA) test for three groups and more. Statistical differences were considered significant at a p value of less than 0.05.

## Additional Information

**How to cite this article**: Boriushkin, E. *et al.* p58^IPK^ suppresses NLRP3 inflammasome activation and IL-1β production via inhibition of PKR in macrophages. *Sci. Rep.* **6**, 25013; doi: 10.1038/srep25013 (2016).

## Supplementary Material

Supplementary Information
