# Supplementary material for: p58IPK suppresses NLRP3 inflammasome activation and IL-1β production via inhibition of PKR in macrophages
Source: Sci Rep. 2016 Apr 26;6:25013. doi: 10.1038/srep25013 (PMC4845006; doi:10.1038/srep25013)
Supplement: Supplementary Information [file srep25013-s1.pdf]

Supplementary information

p58IPK suppresses NLRP3 inflammasome activation and IL-1β production via inhibition of PKR in macrophages

Evgenii Boriushkin[1, 2], Joshua J. Wang [1, 2], Junhua Li[1, 2], Maulasri Bhatta[1, 2], and Sarah X. Zhang[1, 2, 3]

1. Department of Ophthalmology/Ross Eye Institute, University at Buffalo, the State University of New York, Buffalo, NY 14214, USA

2. SUNY Eye Institute, the State University of New York, Buffalo, NY 14214, USA

3. Department of Biochemistry, University at Buffalo, the State University of New York, Buffalo, NY 14214, USA

Corresponding Author:

Dr. Sarah X. Zhang
Department of Ophthalmology/Ross Eye Institute
University at Buffalo
The State University of New York
3435 Main Street Buffalo, NY 14214
E-mail: xzhang38@buffalo.edu

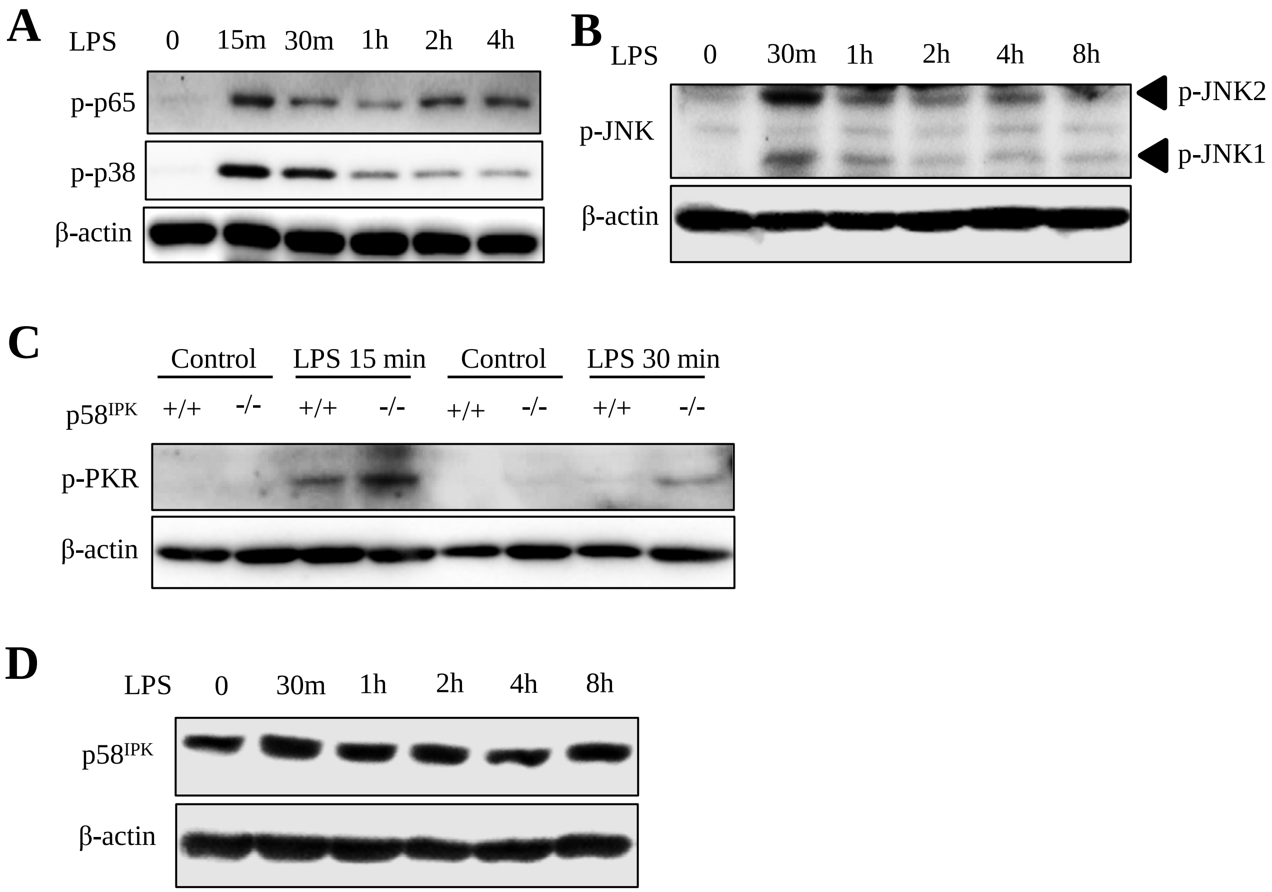

Suppl. Fig. 1. Time course analyses of activation of TLR4 signaling and expression of P58^{IPK} in BMDM after LPS simulation. BMDM isolated form WT mice **(A, B, D)** and p58^{IPK} KO mice **(C)** were treated with LPS (250 ng/ml) for indicated time periods. Protein levels of p-p65, p-p38, p-JNK, p-PKR and p58IPK were evaluated by Western blotting.

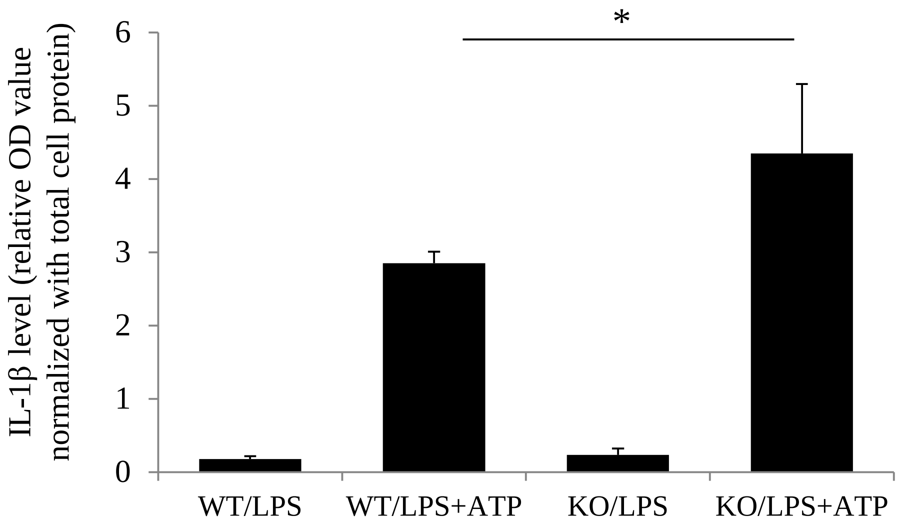

Suppl. Fig. 2. IL-1β secretion from peritoneal macrophages of WT and heterozygous p58^{IPK} KO mice. Primary peritoneal macrophages isolated from WT or heterozygous p58^{IPK} KO mice were stimulated with LPS (500 ng/ml) for 8 h, and then treated with ATP (5 mM) for 1 h to induce inflammasome activation. Cell culture supernatants were collected and IL-1β level in the supernatants were measured by ELISA and normalized with total cell protein. Data are presented as mean ± SD (n=3). * $p<0.05$ (One-way ANOVA).

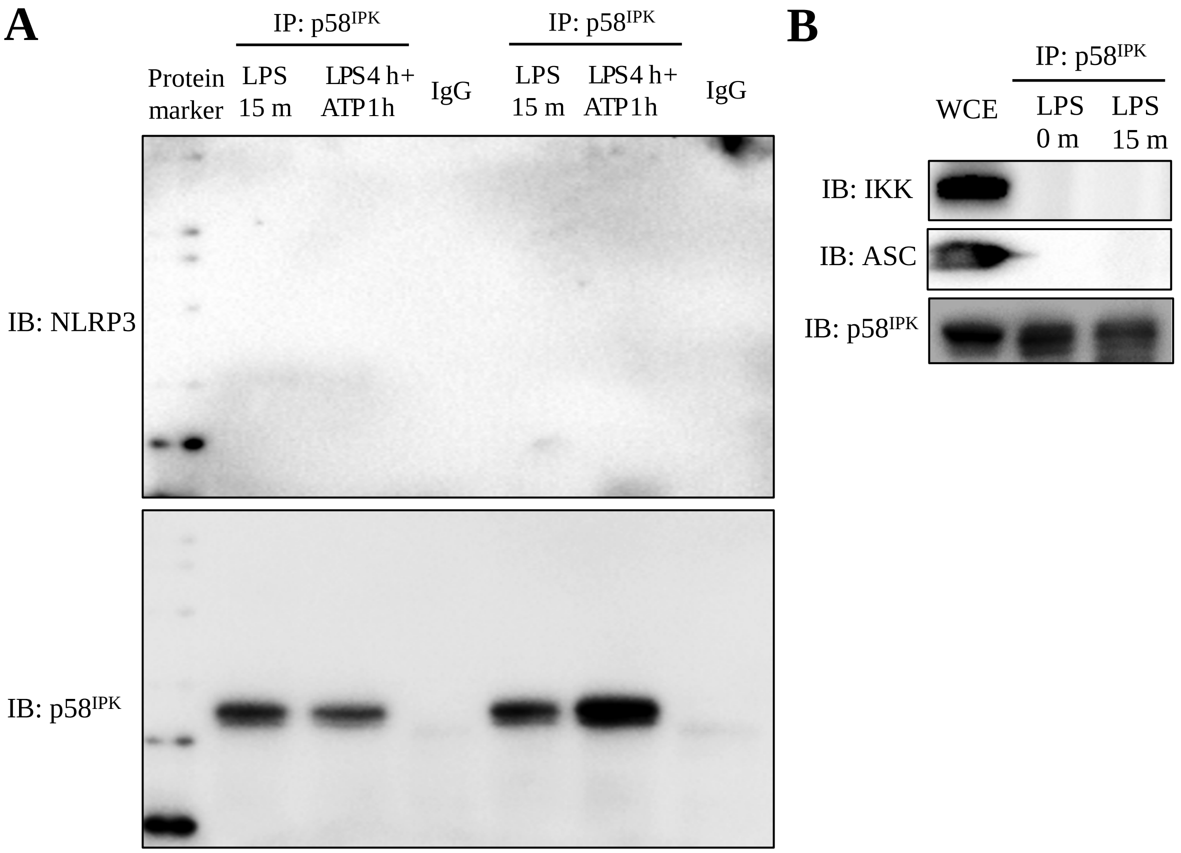

Suppl. Fig. 3. Co-IP analyses showing no positive binding between p58^{IPK} and NLRP3, IKK, or ASC in BMDM before or after indicated LPS treatment. WCE: whole cell extract from BMDM treated with LPS for 15 m.

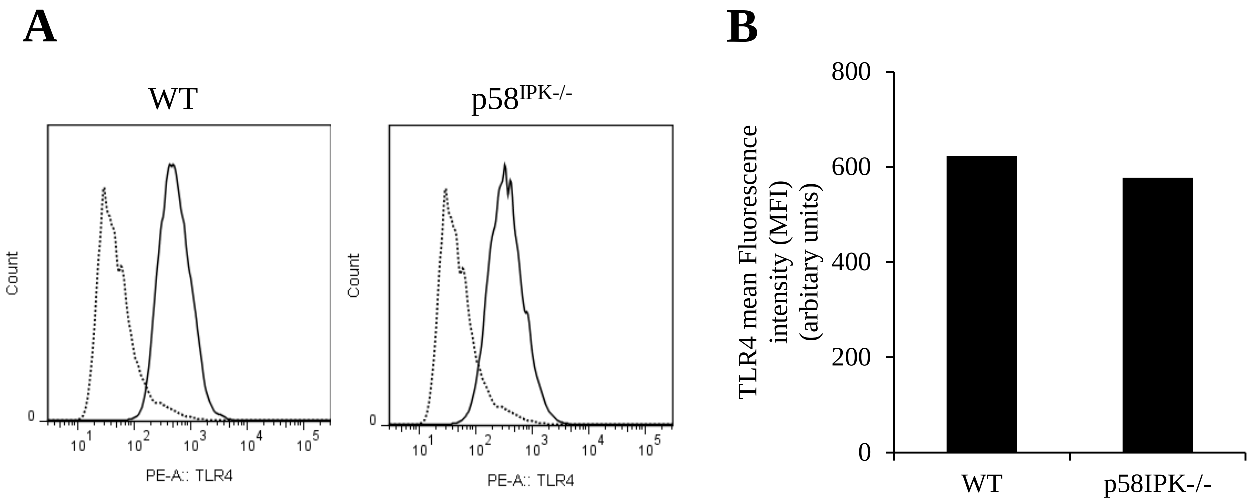

Suppl. Fig. 4. Flow cytometric quantification of TLR4/MD2 expression in WT and p58$^{IPK-/-}$ BMDM. **A.** Flow cytometry histograms of expression of TLR4/MD2 in WT and p58$^{IPK-/-}$ BMDM (dotted line: unstained control; solid line: PE-stained TLR4/MD2). **B.** Quantification of the expression level of TLR4/MD2 based on the mean fluorescence intensity (MFI) in WT and p58$^{IPK-/-}$ BMDM.

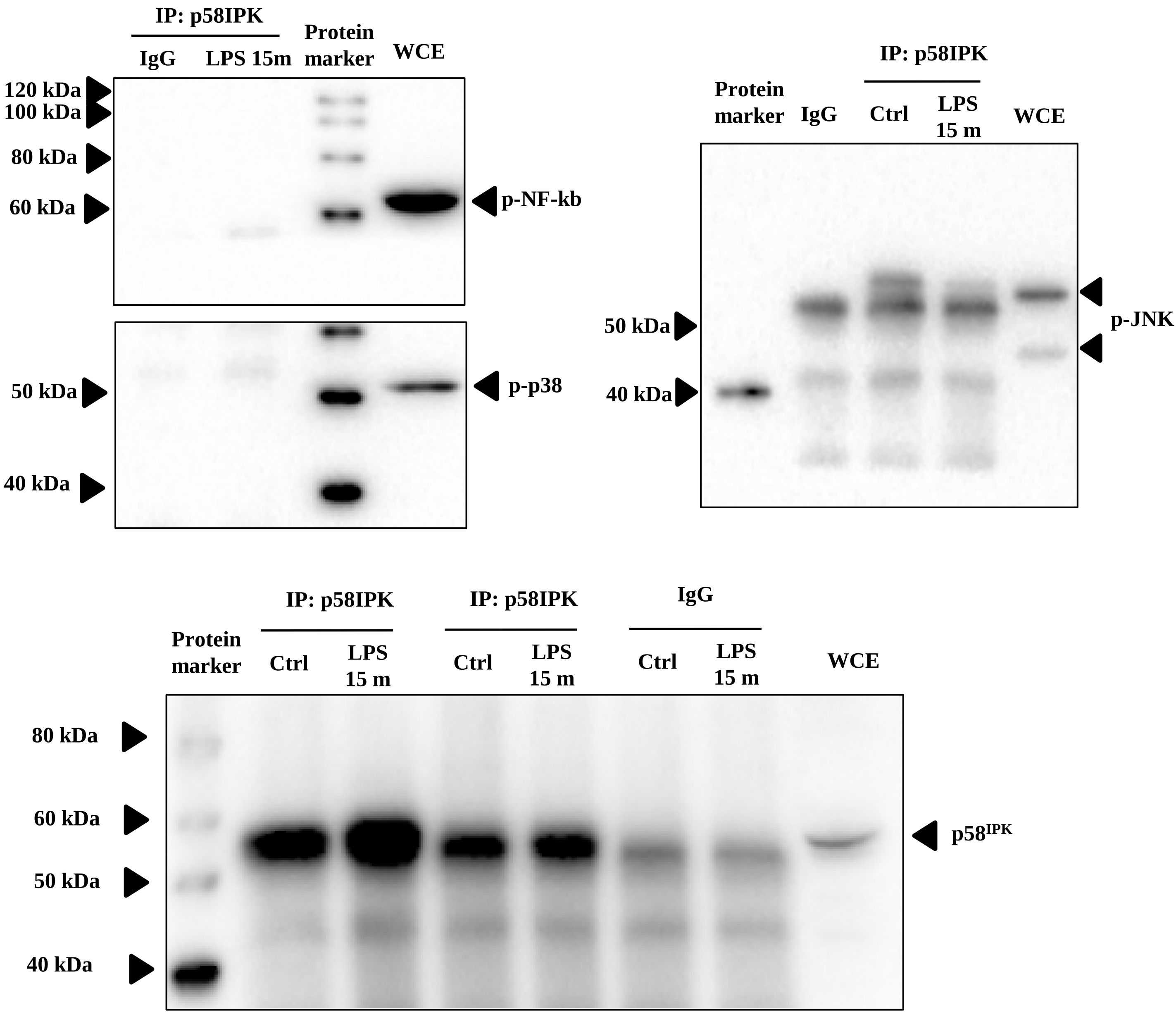

Suppl. Fig. 5. Full-length blots for Figure 5B. Whole cell extract (WCE) from BMDM treated with LPS for 15 min was used as positive control of western blotting. The gels were run under the same experimental conditions.
